# Limited Transfer of Newly Acquired Movement Patterns across Walking and Running in Humans

**DOI:** 10.1371/journal.pone.0046349

**Published:** 2012-09-27

**Authors:** Tetsuya Ogawa, Noritaka Kawashima, Toru Ogata, Kimitaka Nakazawa

**Affiliations:** 1 Department of Rehabilitation for the Movement Functions, Research Institute, National Rehabilitation Center for Persons with Disabilities, Namiki, Tokorozawa, Saitama, Japan; 2 Graduate School of Arts and Sciences, The University of Tokyo, Komaba, Meguro, Tokyo, Japan; Universidad Europea de Madrid, Spain

## Abstract

The two major modes of locomotion in humans, walking and running, may be regarded as a function of different speed (walking as slower and running as faster). Recent results using motor learning tasks in humans, as well as more direct evidence from animal models, advocate for independence in the neural control mechanisms underlying different locomotion tasks. In the current study, we investigated the possible independence of the neural mechanisms underlying human walking and running. Subjects were tested on a split-belt treadmill and adapted to walking or running on an asymmetrically driven treadmill surface. Despite the acquisition of asymmetrical movement patterns in the respective modes, the emergence of asymmetrical movement patterns in the subsequent trials was evident only within the same modes (walking after learning to walk and running after learning to run) and only partial in the opposite modes (walking after learning to run and running after learning to walk) (thus transferred only limitedly across the modes). Further, the storage of the acquired movement pattern in each mode was maintained independently of the opposite mode. Combined, these results provide indirect evidence for independence in the neural control mechanisms underlying the two locomotive modes.

## Introduction

In everyday life, humans use two major modes of locomotion: walking and running. By definition, walking is known as a movement in which at least one foot is always in contact with the ground, whereas running involves aerial phases where both feet are off the ground. Both similarities and dissimilarities between the modes have been demonstrated from the perspectives of energetics [1], limb movements [2], [3], and muscle functions [2], [4], [5]. Because of the spontaneous behavior to transit into the opposite modes in accordance with changing speed (walk-run or run-walk transition) [2], [6]–[8], these two movement modes seem dependent on the demand for different locomotion speeds.

On the other hand, by referring to earlier studies focusing on the behavioral aspect of human motion in simple upper-limb movements [9], [10] and gait [11], [12], neural control mechanisms underlying human movement are considered as very specific to given tasks or contexts. Combined with direct evidence obtained in animal models [13], [14], there would be a possible independency in the neural mechanisms specific to different modes of locomotion. Walking and running in humans therefore, may not only be dependent on different speeds but also have discrete control mechanisms capable of the respective modes. The present study addressed the possibility by utilizing motor adaptation paradigms that have been well established in the field of motor control, especially in the last decade [9]–[12].

Based on the hypothesis that independent neural control mechanisms underlie walking and running, we established working hypotheses as follows. 1) After the acquisition of a novel movement pattern (adaptation) in one of the modes, the emergence of the novel pattern in the subsequent trials is evident only within the same mode and limited in the opposite mode (thus, limited transfer across walking and running). In addition, 2) storage of the novel movement pattern in the respective mode is maintained independently of the opposite mode. The acceptance of these working hypotheses will provide indirect evidence of independent neural mechanisms underlying human walking and running. A section of the results in the present study have been presented in abstract form [15].

## Methods

### Subjects

**Figure 1:**
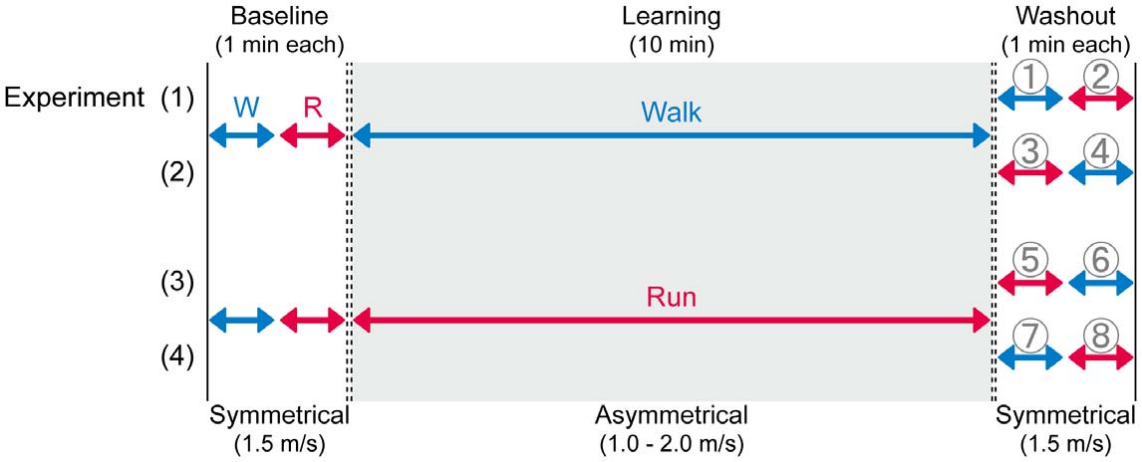
Experimental protocols (1 through 4) adopted in the present study. Subjects underwent adaptation tasks of either walking (1 and 2) or running (3 and 4) on an asymmetrically driven treadmill (one belt was set at 1.0 and the other at 2.0 m s^−1^) for 10 minutes. Walking and running patterns on a normally operated treadmill (at 1.5 m s^−1^ bilaterally and 1 minute each in duration) before and after the adaptation were compared on the basis of the modes of adaptation.

Twenty-four healthy male volunteers (age range, 22 to 49 years old) with no known history of neurological or orthopedic disorders participated in the study. Each subject was tested in two of four experimental protocols (Figure 1). Twelve of them participated in experiments 1 and 2, while the other 12 participated in experiments 3 and 4. The order of participation was randomized across subjects.

### Ethics Statement

Each subject gave written informed consent for his participation in the study. The experimental procedures were approved by the local ethics committee of the National Rehabilitation Center for Persons with Disabilities, Japan, and were conducted in accordance with the Declaration of Helsinki.

### Experiment

In the present study, the subjects walked and ran on a split-belt treadmill (Bertec, Columbus, OH, USA), having two belts (one underneath each foot), each driven by an independent motor. The treadmill was operated either symmetrically (both belts moving at the same velocity) or asymmetrically (at different velocities). During the baseline period, the treadmill was operated symmetrically and the velocity was adjusted to 1.5 m s^−1^. This was the speed where all the subjects could both walk and run comfortably in our pilot experiment. Subsequently, the subjects learned to walk (experiments 1 and 2) or run (experiments 3 and 4) on an asymmetrically driven treadmill for 10 minutes. The speed of one belt was increased by one third from the baseline (0.5 m s^−1^), whereas that of the other was decreased by one third; thus, the belt speeds were 2.0 and 1.0 m s^−1^, respectively. The direction of speed change (either faster or slower) was randomized across subjects and the experimental protocol. After the 10-minute adaptation period, the belt speed was returned to symmetry (for the washout periods) as in the baseline periods. Here, the subjects were instructed to walk and run (experiments 1 and 4) or run and walk (experiments 2 and 3) in order for 1 minute each in duration depending on the experimental protocols (Figure 1). Between all testing periods (baseline walk, run, adaptation, washout walk (run), and run (walk)), the treadmill was stopped once and restarted immediately by the experimenter with an acceleration (deceleration) of 0.5 m s^−2^. The subjects were instructed to walk or run normally as they looked at a wall approximately 5 meters in front of them and were instructed to refrain from looking down at the treadmill belts in order to avoid any visual biases on the speed. The subjects were also instructed to always start their task by either walking or running from the first step depending on the testing sessions. For safety, one experimenter always stood by the treadmill during the experiment, and the subjects could hold onto handrails mounted on both side of the treadmill in case of risk of falling. However, all the subjects satisfactorily completed the testing sessions without using the handrails.

### Recordings and Analysis

Three orthogonal ground reaction force (GRF) components (mediolateral (Fx), anteroposterior (Fy), and vertical (Fz)) were detected by two force plates mounted underneath each treadmill belt. The force data were low-pass filtered at 5 Hz and were digitized at a sampling frequency of 1 kHz (Power Lab, AD Instruments, Sydney, Australia). From the Fz component of the GRF, the moments of ground contact and toe-off were detected on a stride-to-stride basis using a custom-written program (VEE pro 9.0, Agilent Technologies, Santa Clara, CA, USA). Data on the first stride cycle of each testing session were removed for later analysis in order to minimize the influences of perturbation induced by the initiation of the treadmill movements.

The aspects of walking and running were investigated by addressing the peak anterior braking force upon foot contact for every stride cycle. In our pilot study, we demonstrated that, among all of the orthogonal ground reaction force (GRF) components, only this component showed clear aspects of adaptation and aftereffects with the return to symmetrical belt condition in both walking and running. A series of previous studies focused on temporal and spatial gait parameters such as stride and step length, stance and swing time, double support time, and the relationship in the gait phase between the two legs to address adaptive behavior of the split-belt treadmill walking [11], [12], [16], [17]. However, given that gait speed is a quotient of length (spatial) and the time (temporal factors), subjects could potentially employ different strategies across individuals (either walking or running with spatially symmetrical with temporally asymmetrical movement patters, temporally symmetrical with spatially asymmetrical movement patterns, or changing the both parameters) with exposure to belt conditions with changing speed.

Since the stride cycles taken during the testing sessions varied across subjects and tasks (walk or run), the obtained data were averaged over stride cycles in 3-second bins and were normalized to the mean during the baseline of each movement task (walk or run) to allow intersubject comparisons.

For statistical comparisons, two-way analysis of variance (ANOVA) with repeated measures was used to test for statistically significant differences in the aftereffects, with factors of movement modes (walk or run) or the previously imposed adaptation tasks and the time in the respective 60-second washout period. Data are presented as the mean and standard error of the mean (mean±SEM). Significance was accepted when P<0.05.

## Results

The number of stride cycles taken under the identical speed differed depending on the movement mode and among subjects. Regardless of the belt condition (symmetric at 1.5 m s^−1^ or asymmetric at 1.0 m s^−1^ and 2.0 m s^−1^), subjects on average took approximately 60 stride cycles for walking and 80 strides for running every minute.

**Figure 2:**
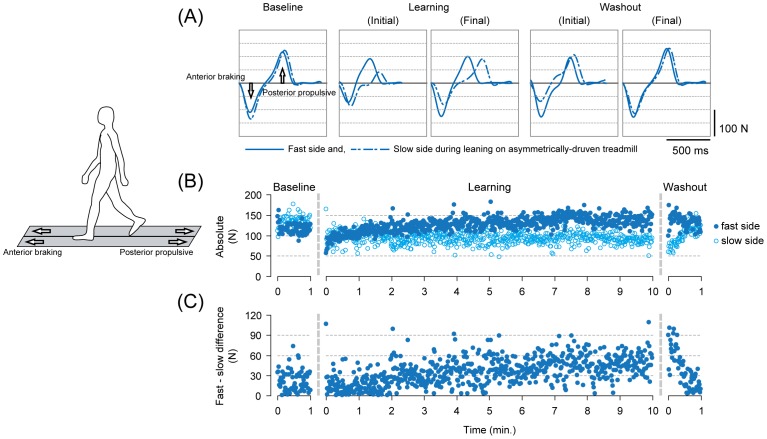
Descriptions of adaptation on the asymmetrically driven treadmill and the emergence of the aftereffect with release from the novel environment in walking in a single subject (showing only the walking periods from Experiment 1). (A) Waveforms of the antero-posterior ground reaction force under different time points in the experiment. Each waveform represents an ensemble average of five consecutive stride cycles (from heel contact to the subsequent heel contact) in the respective time points. The solid lines represent the fast-moving side and the dotted lines are those of the slow side during the adaptation period. (B) Stride-to-stride profile of the peak anterior braking force for both fast and slow sides. Filled circles and open circles represent the fast and slow sides, respectively. (C) Stride-to-stride profile of the differences in peak anterior braking force between the fast and slow sides.

**Figure 3:**
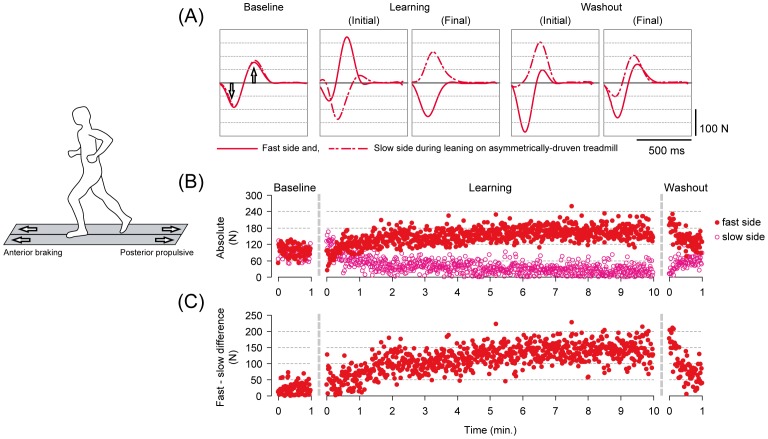
Descriptions of adaptation on the asymmetrically driven treadmill and the emergence of an aftereffect with release from the novel environment in running in a single subject (only the running periods from Experiment 3 are shown). (A) Waveforms of the antero-posterior ground reaction force under different time points in the experiment. Each waveform represents an ensemble average of five consecutive stride cycles (from heel contact to the subsequent heel contact) in the respective time points. The solid lines represent the fast-moving side and the dotted lines are those of the slow side during the adaptation period. (B) Stride-to-stride profile of the peak anterior braking force for both fast and slow sides. Filled circles and open circles represent the fast and slow sides, respectively. (C) Stride-to-stride profile of the differences in peak anterior braking force between the fast and the slow sides.

All of the subjects reported that their movement patterns were disturbed when returning to the symmetrical belt conditions after walking on the asymmetrically driven treadmill, as described in previous studies [11], [16]. For running after adapting to run on asymmetrical belts, subjects also reported their movement patterns as perturbed. Figures 2 and 3, respectively, show typical examples of antero-posterior (braking and propulsion, respectively) ground reaction force waveforms under different time points (A), time series changes in the peak anterior force for both fast and slow sides (B), and the differences in the peak force between the sides (C) on a stride-to-stride basis for walking (Figure 2) and running (Figure 3).

During the baseline where the belt conditions were symmetrical, the waveforms were very similar in shape and the amplitude (both anterior and posterior components) between the sides for both walking (Figure 2 (A)) and running (Figure 3 (A)). With exposure to the asymmetrical belt condition, the shapes resulted in prominent differences, an indication of different movement patterns between the fast and the slow sides. For both walking and running, modification in the amplitude of peak anterior braking force took place in the 10-minutes learning periods, including both rapid changes in the earlier phase (up to around 1 minute) followed by slower gradual changes (Figure 2 (B) and Figure 3 (B)). The modification in the amplitude was an increment for the fast side and a decrement for the slow side, respectively. It is especially noticeable here that the braking force in the slow side almost disappeared at the fully adapted state in running (Final of Learning period in Figure 3 (A) and near 10 minutes in the Learning period in Figure 3 (B)). As a consequence, there were large differences between the sides (asymmetry) (Figure 2 (C) and Figure 3 (C)). With return to the symmetrical belt condition (washout), the amplitudes of the force differed to a great extent between the sides despite the identical belt speed to that during the baseline. In detail, there were initially an overshoot in the amplitude for the fast side and an undershoot in the slow side for both walking and running (in comparison to the baseline). In the 1-minute washout period, the amplitudes of both sides decayed toward those found in the baseline (into the opposite direction to the changes during the learning periods). An important fact here is that the movements were initially disturbed upon walking on symmetrical belt after adapting to walk, and running after adapting to run, on the asymmetrically driven treadmill surface. The disturbance in the movements were then, followed by gradual decay (restoring normal movements) in the following 1 minute.

It should be noted that modification in the force occurred in the posterior (propulsive) component as well. In the representative waveform (Figure 3 (A)), for example, the posterior force in the fast side showed a sudden increase with exposure to the asymmetrical belt but subsequently disappeared at the end of the learning period. Combined with that in the slow side which showed a modification into the opposite direction (increase), there was large asymmetry at the initial state of the washout period. The asymmetry, however, was prominent only in running and not in walking. We therefore used anterior braking force (disturbed both in walking and running) as parameter in the present study.

**Figure 4:**
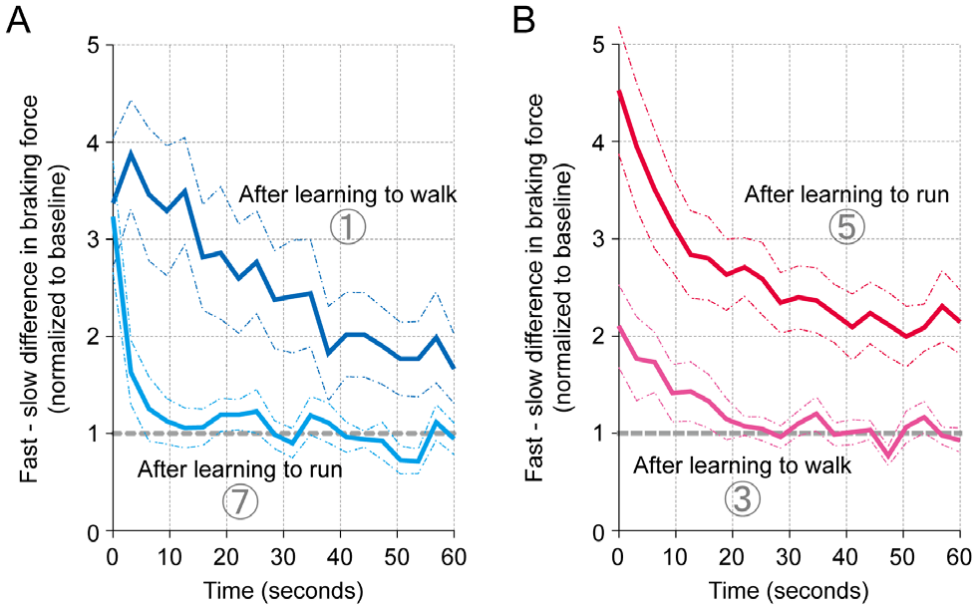
Degree of transfer in the acquired movement pattern across walking and running, shown as differences in the peak braking force between the sides. The extent of asymmetry in (A) walking after adaptation to walk (first washout period in Experiment 1, darker line) and after adaptation to run (first washout period in Experiment 4, lighter line), and (B) running after adaptation to run (first washout period in Experiment 3, darker line) and after adaptation to run (first washout period in Experiment 2, lighter line). Data are normalized to the mean of those during the baseline on a subject-to-subject basis and are presented as the mean (thick line) and the standard errors of the mean (dotted lines).

Given the initial disturbance in the movement patterns (asymmetry in the braking force) in both movement modes after adapting in each mode, the primary interest in the present study was whether the movement pattern acquired through each mode transferred to (or shared with) the other mode. Figure 4 (A) compares the extent of asymmetry in walking on identical belt conditions after adapting to walk (blue line) and after adapting to run (light blue line) as differences in the peak force between the sides. In contrast to the large asymmetry after learning to walk, the emergence of aftereffect was only partial (only reactively present in the first few seconds). ANOVA comparison revealed a significant difference between walking with different history (learned to walk or run) in previously imposed adaptation modes (F_1, 22_ = 7.285, P<0.05). On the other hand, the degree of aftereffect during running with a different adaptation history is described in Figure 4 (B). In comparison to the prominent asymmetry in the running patterns after adapting to run, individuals who adapted to walk showed far less asymmetry (F_1, 22_ = 15.914, P<0.01).

**Figure 5:**
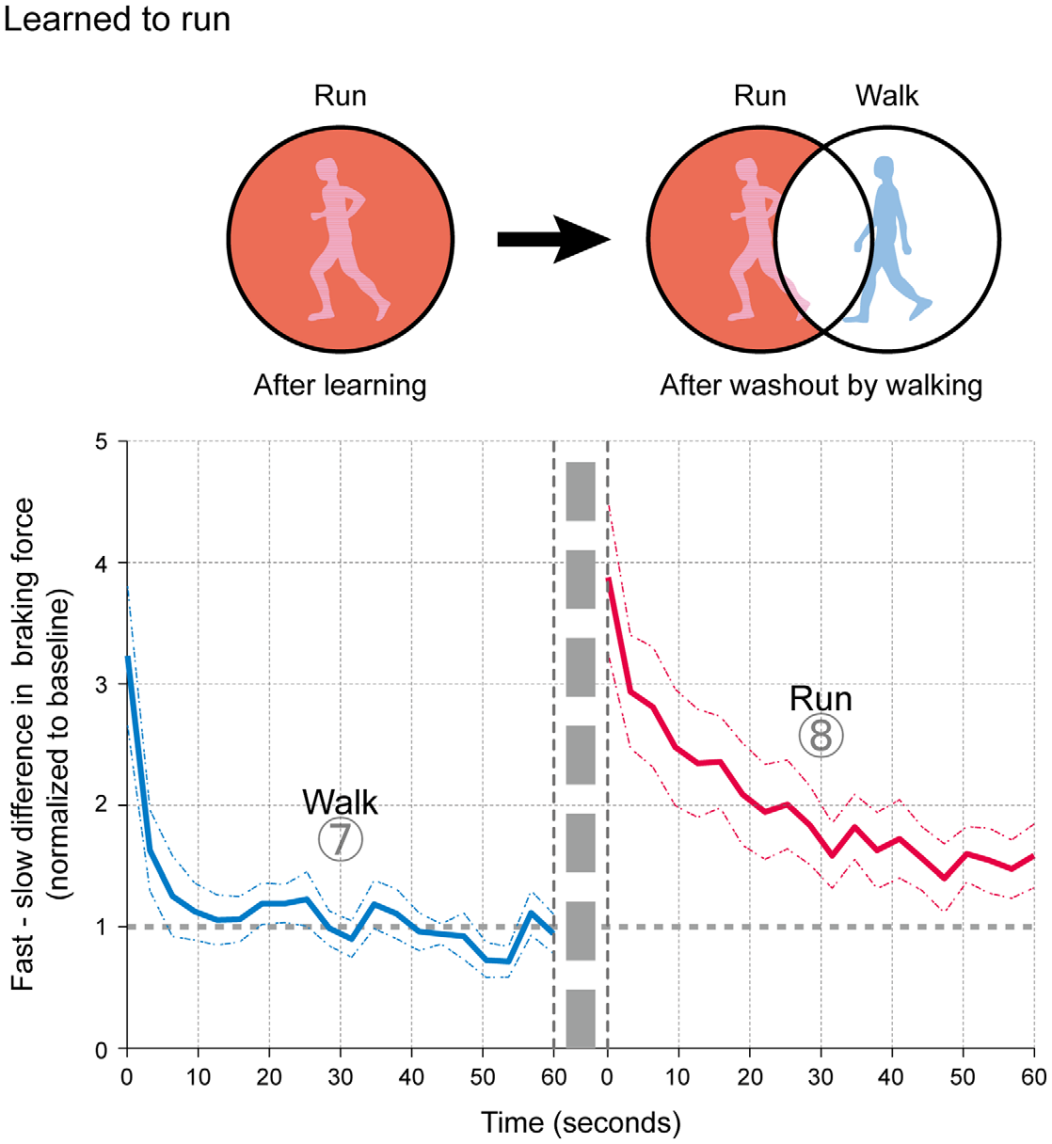
Degree of washout in the stored motor pattern in running by walking (first and second washout periods shown consecutively from Experiment 4). The asymmetrical movement pattern was evident with the initiation of running (red lines) despite a symmetrical walking pattern at the end of the first washout period in walking (blue lines), an indication of only partial washout (also described in the schematic figure). Data are presented as means (thick lines) and their standard errors of the mean (dotted lines).

**Figure 6:**
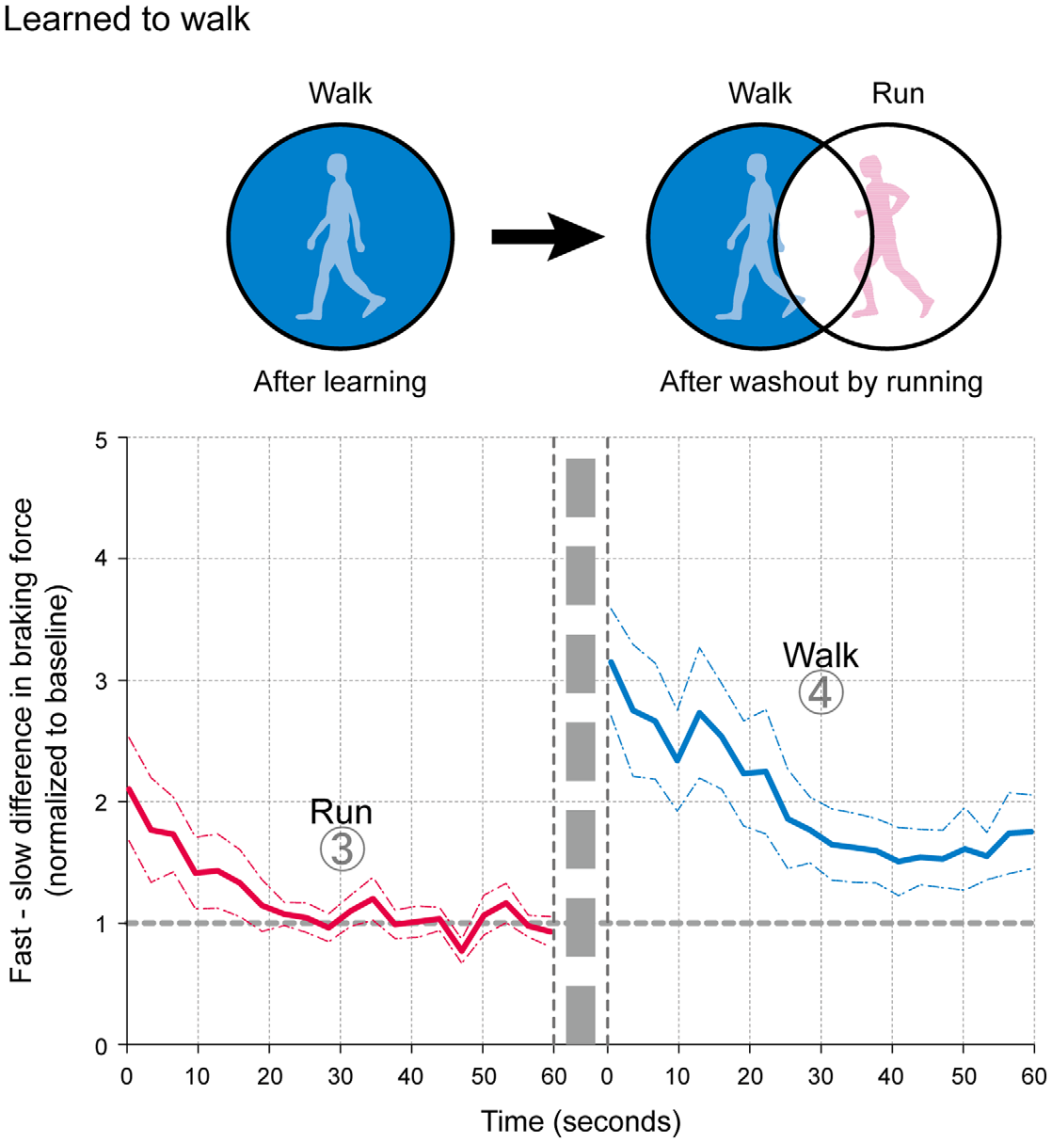
Degree of washout in the stored motor pattern in walking by running (first and second washout periods shown consecutively from Experiment 2). The asymmetrical movement pattern was evident with the initiation of walking (blue lines) despite the symmetrical walking pattern at the end of the first washout period in running (red lines), an indication of only partial washout (also described in the schematic figure). Data are presented as means (thick lines) and their standard errors of the mean (dotted lines).

Secondly, to further consider the independence or commonality of each movement mode in relation to the other, we investigated the extent of a possible washout in the acquired movement patterns in one mode by the other (Figures 5 and 6). As partially described in the results above, the subjects could both walk and run as normal at the end of the first washout period after adapting in the opposite modes (shown in the left columns in Figures 5 and 6). The subsequent attempts to run (right column, Figure 5) and walk (Figure 6) resulted in prominent asymmetry in the movement patterns, demonstrating little or no washout by the execution of the opposite mode. That is, the acquired movement patterns (asymmetry) were maintained independently of the subsequent trials in the opposite modes. ANOVA showed significant differences in the degree of asymmetry in the movement patterns between the first and second washout periods (F_1, 11_ = 6.109, P<0.05, for 1) walking, and 2) running after adapting to run (F_1, 11_ = 6.914, P<0.05, for 1) run and 2) walk after adapting to walk).

## Discussion

The present results strongly confirmed our working hypotheses and demonstrated that 1) transfer of the novel movement patterns learned on an asymmetrically driven treadmill from one mode to another took place only partially for both directions (walk to run and run to walk), and 2) the learned movement patterns in the respective modes were rarely washed out by the subsequent execution in the opposite modes, again, for both directions. That is, the storage of a learned movement patterns were maintained independently of the opposite mode. Combined, these results demonstrated only partially overlapped elements between these two movement modes and thus support the notion of mostly independent functional networks within the CNS for the respective locomotive modes. Walking and running, therefore, reflect not only functions of different speeds of locomotion, but are different forms from the perspective of neural control mechanisms.

The notion of task-specific or context-specific neural mechanisms has been well established by using simple reaching movements in the upper extremities [9], [10]. Locomotive movements that are more complex and autonomic have also been found as under the specificity, such as the direction (forward-backward) [11], the limb (right-left) [11], and the speed of walking [12]. Limitations in the transfer or washout in newly acquired movement patterns under certain physical constraints in one movement tasks to or by another have been accepted as indirect evidence demonstrating the specificity [9]–[12]. By adopting the well-established experimental paradigms in the earlier studies, the present study is the first to address the mode-specificity, comprising an important aspect of locomotion. Because of the well-known spontaneous behavior to transit into the opposite mode (walk-run or run-walk transition) in accordance with changing speed [2], [6]–[8], walking and running may only be considered as a function of demands for different speeds.

The use of split-treadmill walking to modify gait symmetry has been studied extensively in the last decade [11], [12], [6]. After walking on an asymmetrically-driven treadmill for a certain period of time, the movement pattern after release from the novel environment resulted in prominent asymmetry [11], [12], [16]. The current study, for the first time, demonstrated that movement patterns in running also could be modified as in the earlier studies focusing on walking. Detailed explanations on how the gait patterns could be adapted with exposure to the asymmetrically driven treadmill and resulted in the subsequent aftereffect have been provided previously both behaviorally and mathematically on the basis of locomotion in decerebrate cat [18].

In the present study, the modification in the gait patterns was most evident in the anterior braking component of the ground reaction force both in walking and running and we therefore focused on this parameter (detailed description in the Methods). As subjects adapted to walk or run comfortably on the asymmetrically driven treadmill, the patterns of modification in the anterior braking force showed gradual increment in the fast side and decrement in the slow side, both including brief and more rapid changes in the early phases of exposure. As a consequence, with return to the symmetrical belt in the washout period, there was initially an overshoot in the force in the fast side and an undershoot for the slow side, both followed by gradual decay into the opposite direction to those during the adaptation periods (towards baseline). Combined with results in a previous study in which novel motor pattern could be stored intralimb and independently for each leg [11], these phenomena occurring for the each limb may reflect the well-established notion of motor adaptation or learning where motor output is recalibrated to meet new task demands [19]. It is reasonable to consider that the asymmetry in the anterior braking force took place based on the recalibration of motor output in each leg under different velocity on an asymmetrically driven treadmill.

The motor output acquired through the above mentioned recalibration processes, however, were only partially shared across the movement modes. Given the results, with the possibility of specificity in the neural mechanisms underlying walking and running, the discussion will now focus on the possible neural mechanisms comprising the different modes. Based on the results of animal studies and of humans, the neural mechanisms underlying the present results could be attributed to possible contribution of supraspinal structures in the brain and the specificity in the locomotor center in the spinal cord, known as the central pattern generator (CPG).

First, in the emergence of the adaptive phenomena, the cerebellum is considered to play a significant role by recalibrating motor output that satisfies the task or environmental demand [20]. Given its function, any aspect of an aftereffect following adaptation is abolished in humans [17] and in cats [21] with cerebellar lesions. Morton et al. (2006) [17] reported that a predictive feedforward motor adaptation in splitbelt treadmill walking that is demonstrated to occur in healthy subjects [11], [12], [16] does not in patients with cerebellar damage. More direct evidence showed that plasticity of synaptic transmission efficacy in the cerebellum that was modified by concentration of nitric oxide (NO) played a significant role in locomotive adaptation in decerebrate cat [21]. Interestingly, regarding movement specificity, various aspects of limb movement such as direction, velocity, acceleration and force have been demonstrated to be represented in the cerebellum, as shown by discharge rate in single unit recording in the cerebellum [22]. In the present study, since the subjects performed both walking and running under identical belt speed, in which the limb movements do not simply depend on locomotion speed but are demonstrated to differ across the modes [3], it is possible that there were different representation for each locomotive mode.

Along with the cerebellar function, the contribution of the descending neural drive from the supraspinal centers, especially those from the mesencephalic locomotor region (MLR) in the brainstem, provides an additional explanation for the mode-specificity. For example, in decerebrate salamander, electrical microstimulation at a particular site in the MLR resulted in a phase-dependent electromyographic (EMG) burst and consequently locomotor-like movements of the body [23]. In the emergence of these behaviors, two different locomotor modes (stepping and swimming) were exhibited with different current intensities [23]. Or, more classically, an increase in stimulus intensity to the mid-brain in decerebrate cats walking on a treadmill caused them to gallop [24]. From these results, the intensities in the descending drive may significantly affect the decision of different locomotive modes. In the current study, although speculative, the gait pattern upon the initiation of walking after adapting to run was reactively disturbed (the prominent asymmetry in the first few seconds, shown by the light blue line in Figure 4). This reaction may reflect the component of running. That is, to accelerate the center of body mass upon acceleration of the treadmill by increasing the descending drive from the locomotor centers. Consequently, this could result in the partial emergence of the asymmetrical movement pattern previously acquired in running.

Regarding the specificity in the locomotor center in the spinal cord, on the other hand, it was recently demonstrated that specific sets of spinal interneurons are activated depending on locomotion (swimming) frequency in larval zebrafish [14]. Locomotion behavior in larval zebrafish was previously characterized as having two different modes [25]. One is the mode used to move routinely in water with lower movement frequencies and small yaw amplitudes, while the other is the escape movement with higher frequencies with larger yaws [25]. On the execution of these locomotor behaviors by zebrafish, McLean et al. (2008) [14] showed that, in contrast to motoneurons that are additionally recruited with increasing swimming frequencies following classic size principle, the activities in some sets of interneurons evident under lower swimming frequency were inhibited during swimming at higher frequencies [14]. In other animal models, such as in a fictive scratching movement in the turtle hindlimb, it was found that different populations of propriospinal neurons were identified with respect to two different modes of scratching movements [13]. Based on these previous results in animal models, it is speculated that the specific structures to be selected in the spinal cord depending on the modes might explain the underlying differences in the neural mechanisms between walking and running in humans.

Regarding adaptation as observed in the present study and in previous studies [11 12,16], the spinal cord itself is known to be capable of adapting locomotor patterns, as predominantly demonstrated in the stepping movement of human infants [26] or in cats that underwent complete spinal cord transection [27]. The relationship between mode specificity and adaptation remains unclear. It is however, reasonable to consider that the acquisition of the novel movement patterns took place in particular sites in the spinal cord or in combination with the higher structures depending on the mode, at least before motoneuron, which is the final common pathway to muscles. The acquired movement patterns were therefore only partially transferred to the opposite modes, which have different responsible sites and were rarely washed out by the execution of the opposite ones.

In summary, the two major modes of human locomotion, walking and running, are not only functions of different speed but have fundamentally different neural control mechanisms. The present results provide extremely important implications for the construction of training regimens in locomotive movements in both athletic training and rehabilitation processes. Further considerations should be made among other locomotive tasks or those under different physical constraints.
